# Does pigtail catheters relieve pneumothorax? A PRISMA-compliant systematic review and meta-analysis

**DOI:** 10.1097/MD.0000000000013255

**Published:** 2018-11-21

**Authors:** Ming Fang, Guilin Liu, Guoliang Luo, Tianyu Wu

**Affiliations:** Department of Ultrasound, The Second Affiliated Hospital (Jiande Branch), Medical School of Zhejiang University, The First People's Hospital of Jiande, Hangzhou, China.

**Keywords:** complication, drainage, meta, pigtail catheter, pneumothorax

## Abstract

**Background:**

Pigtail catheter drainage has been usually applied for the treatment of pleural effusion and pneumothorax. Our aim was to investigate the application and efficacy of pigtail catheters for pneumothorax.

**Methods:**

We carried out a meta-analysis of retro- or pro-spective studies addressing the effect of pigtail catheters for pneumothorax. We presented success rates of pigtail catheter drainage as primary outcomes, and considered the duration of drainage, and complication rates as secondary outcomes. Pooled data were available using the fixed or random effects model. Heterogeneity, sensitivity, and subgroup analyses were performed.

**Results:**

The meta-analysis was based on 16 articles with a total of 1067 patients. Our analyses showed that pooled success rates were 0.77 (0.71–0.82), )furthermore, duration of drainage was 5.61 (3.99–7.23), and complication rates 0.18 (0.09–0.27). Subgroup results according to causes of pneumothorax and patient characteristics were robust and all consistent with overall outcomes.

**Conclusion:**

These suggested that pigtail catheter insertion within radiological guidance may provide a safe and effective way for the treatment of pneumothorax. More large-scale and prospective studies were required to determine these findings.

## 1 Introduction

Pigtail catheters (PC) drainage under radiological guidance has became an alternative for pneumothoraxes and pleural effusions.^[1,2]^ There were no studies that identified traditional large-bore chest tubes (16F–32F) were superior to that of small-bore pigtail catheter (8F–14F) in the management of pneumothorax and pleural effusion. British Thoracic Society pleural disease guideline 2010 indicated that the tip of the chest tube or a pigtail catheter should be placed at the top and front of the pleural cavity.^[3]^ Furthermore, large-bore chest tube could easily cause injury of chest wall and the adjacent organs. It was reported that pain, intrapleural infection, wound infection, drain-related visceral injury, and drain blockage are the most common complications caused by large-bore chest drain insertion.^[3]^ However, results varied as to the success rates, duration of drainage and complication reported in different studies.^[4,5]^ Previous studies have shown that smaller tubes induced less pain, but whether the clinical advantage could bring a better clinical outcome was still unknown.^[6]^ Thus we conducted a systematic review and meta-analysis to obtain precise estimates of the safety and efficacy of pigtail catheters in pleural effusion and pneumothorax.

## 2 Methods

### 2.1 Search strategy and study eligibility

In order to explore pigtail catheters and the risk of pneumothorax, the study was performed according to PRISMA Statement.^[7]^ Before June 2018, the following databases were systematically retrieved: PubMed, Embase, Scopus, Web of Knowledge, and Cochrane library. The search was based on the following terms: pigtail catheters, pneumothorax, hematopneumothorax, aeropleura, and aerothorax. The retrieval was limited to the human population with retro- or pro-spective studies.

In addition, due to the meta-analysis, it is not necessary to obtain ethical approval.

### 2.2 Inclusion and exclusion criteria

The eligible studies for this meta-analysis were selected according to the following criteria: they recorded the information of success rates, duration of drainage, and complication. They were published original articles. Exclusion criteria were as follows: they did not contain pigtail catheters in full text. They were not case reports, meeting abstract or letters. After initial retrieval, titles and abstracts were screened for further assessment according to inclusion criteria by 2 researchers.

### 2.3 Data extraction and quality assessment

Two independent reviewers cross-checked and extracted data into tables based on the predefined criteria. Successful outcome was considered as continuous complete or near-complete re-expansion of the lung for >24 hours after therapy.^[8]^ The primary outcomes were success rates, and secondary outcomes included duration of drainage, and complication. Pooled success rates and complications rates were calculated to combine the summary result from each subgroup. We collected and synthesized the mean and standard deviation (mean ± SD) for assessment of duration of drainage through each study. When conflicts occurred during data extraction, they were solved by discussion or a third reviewer. Risk of bias was assessed using the Grading of Recommendations Assessment, Development and Evaluation (GRADE) guideline, considering the randomized controlled trial (RCT) as an initial high quality, which were degraded if existing in the risk of bias, inconsistency, indirectness, imprecision, or publication bias. Otherwise, cohort studies were as an initial low quality, which were upgraded if absent regarding large effect, plausible confounding, or dose–response gradient.^[9]^ Two reviewers independently appraised the risk of bias, and any discrepancies were discussed with a third reviewer. We used the fixed or random effect model to calculate the pooled prevalence estimates and its 95% confidence interval (CI). *I*^2^ statistics, chi-square test (*χ*^2^), and *τ*^2^ were used to reveal statistically between-study heterogeneity. We used Egger test to evaluate *P* value for publication bias when the number is relatively small.^[10]^ Given the potential confounders, we assessed the effect of causes of pneumothorax (spontaneous, secondary, traumatic, and iatrogenic) and patient characteristics (children and adults) in a s predefined subgroup analysis. In addition, we examined the robustness of the meta-analytic results through sensitivity analysis. All analyses were performed using Stata 12.0 software (Stata Corp, College Station, TX). A *P* value of .05 was considered to suggest statistical significance.

## 3 Results

### 3.1 Study characteristics

**Figure 1:**
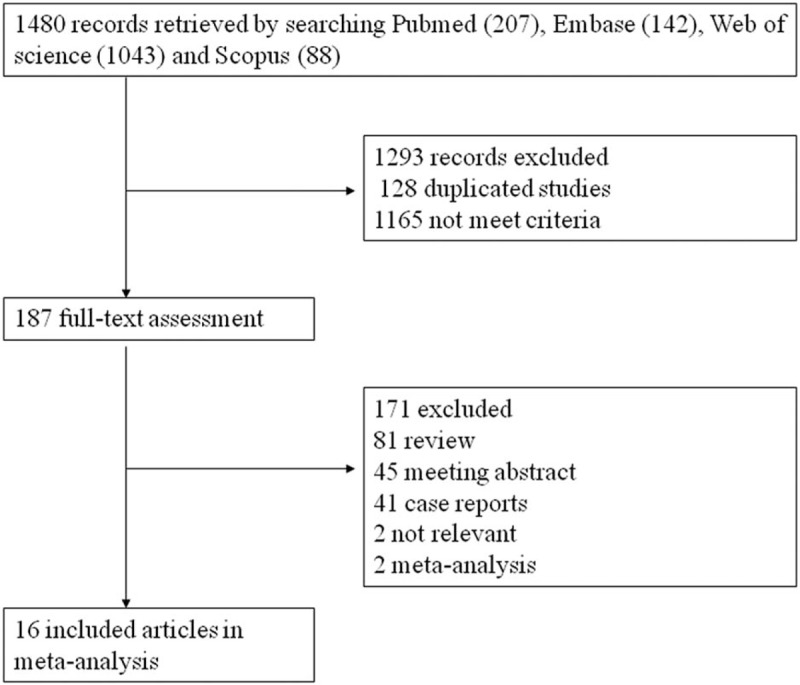
Systematic review flow diagram.

Initially, 1480 articles with pigtail catheters for pneumothorax were screened, and 1294 studies were excluded from further examination (Fig. 1). A total of 1067 patients (776 men and 301 women; age range, 0.77–63.8 years) were enrolled into this study. Table 1 showed the characteristics of the 17 included studies.^[1,2,4–6,11–21]^ Among these articles, 5 were from America, 8 were from China, 1 was from Canada, 1 was from Korea, 1 was from Egypt, and 1 was from Denmark. All the articles were 15 cohort studies and 2 RCTs with mean follow-up ranging from 4 to 36.68 days. During the quality assessment of the studies, 2 RCTs were considered high quality, 13 cohort studies were considered moderate quality, and another cohort study was rated low quality because of the risk of bias.

**Table 1:**
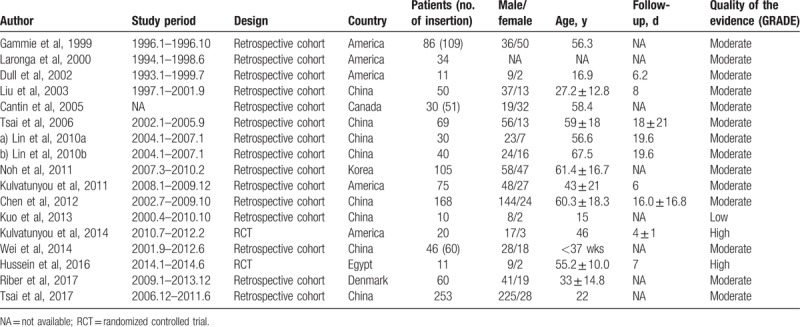
Characteristics of included studies of pigtail catheters for pneumothorax.

### 3.2 Primary and secondary outcomes

**Figure 2:**
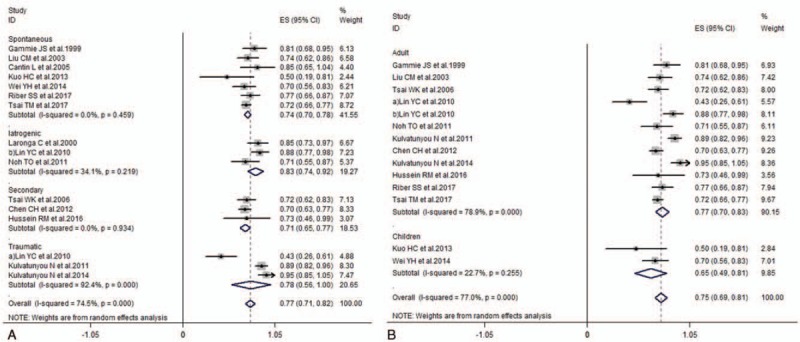
Forest plot of success rates of pigtail catheter for pneumothoraxe according to study design (cohort and RCTs) (A) and race (Caucasian and Non-Caucasian) (B). RCT = randomized controlled trial.

**Figure 3:**
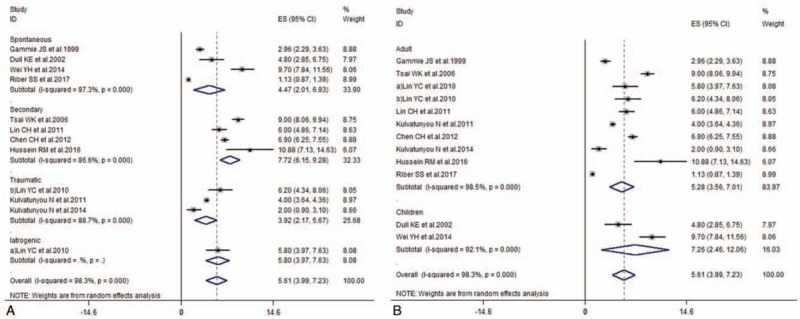
Forest plot of duration of pigtail catheter drainage.

**Figure 4:**
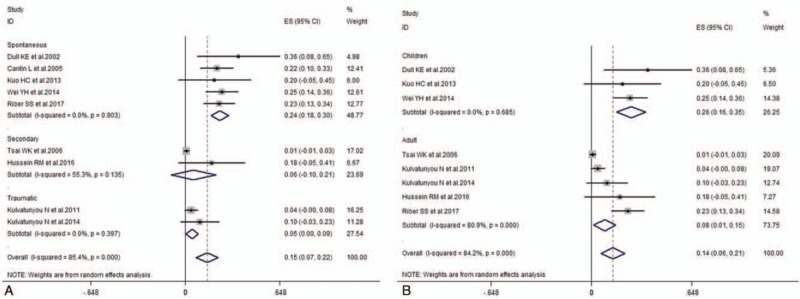
Forest plot of complication rates of pigtail catheter drainage.

Pooled success rates were calculated to be 0.77 (0.71–0.82), and there was obvious heterogeneity (*I*^*2*^ = 74.5%, *P* < .001). In subgroup analysis according to causes of pneumothorax, it showed that success rates were 0.74 (0.70–0.78) in spontaneous, 0.71 (0.65–0.77) in secondary, 0.78 (0.56–1.00) in traumatic, and 0.83 (0.74–0.92) in iatrogenic groups, respectively. Meanwhile, subgroup analysis performed according to patient characteristics, showed that success rates were 0.77 (0.70–0.83) in adult and 0.65 (0.49–0.81) in children, respectively (Fig. 2). The pooled duration of drainage was 5.61 (3.99–7.23) (*I*^*2*^ = 98.3%, *P* < .001) (Fig. 3). In subgroup analysis according to causes of pneumothorax, it showed that duration of drainage was 4.47 (2.01–6.93) in spontaneous, 7.72 (6.15–9.28) in secondary, 3.92 (2.17–5.67) in traumatic, and 5.80 (3.97–7.63) in iatrogenic groups, respectively. Meanwhile, subgroup analysis according to patient characteristics, showed that duration of drainage was 5.28 (3.56–7.01) in adult and 7.26 (2.46–12.06) in children, respectively. Mean complication rates were 0.18 (0.09–0.27) (*I*^2^ = 75.2%, *P* < .001) (Fig. 4). In subgroup analysis according to causes of pneumothorax, it showed that complication rates were 0.24 (0.18–0.30) in spontaneous, 0.06 (–0.10–0.21) in secondary, and 0.05 (0–0.09) in traumatic groups, respectively. Meanwhile, subgroup analysis according to patient characteristics, showed that complication rates were 0.08 (0.01–0.15) in adult and 0.26 (0.16–0.35) in children, respectively.

In addition, there was publication bias observed from Begg test in duration of drainage, and complication (*t* = 2.68, *P* = .023; *t* = 2.94, *P* = .026).

## 4 Discussion

Pneumothorax was still an intractable disease without proper therapy guidelines. In this systematic review and meta-analysis of 1124 cases of pneumothorax from 18 articles, we found a significant benefit of pigtail catheters for pneumothorax. In decades, emerging studies indicated success rates of pigtail catheters drainage ranging from 50% to 98%. In our study, it has shown no significant difference for PC drainage of pneumothorax in spontaneous, secondary, traumatic, and iatrogenic group. Kulvatunyou et al^[16]^ reported that of the PC adults, 89% (67/75) were successfully inserted for traumatic pneumothorax. Gammie et al^[19]^ described a favorable experience with the 8.3F pigtail catheter as a less invasive alternative rather than traditional chest tube insertion, and clinical success rates in the effusion and pneumothorax groups were 86% (66/77) and 81% (26/32), respectively. In a retrospective study, pigtail catheter drainage was successful in 71.9% (182/253) of patients.^[1]^ Chen et al^[20]^ found that pigtail catheter drainage was suitable as an initial management for adults with secondary pneumothorax associated with obstructive lung conditions and malignancy. These indicated that PC has a favorable success rates of pneumothorax.

The main complications of pigtail catheter drainage are pneumothorax, hemorrhage, and chest pains. Dull and Fleisher^[6]^ found no major complications in the PC group consisting of 69 cases. There were 3 insertion-related complications occurred in 75 patients using PC placement over a 2-year period.^[16]^ However, in chest tube drainage, it reported that the frequency of complications was 54.5% (6/11).^[18]^ Another study also found 17.4% (4/23) patients from children experienced minor complications.^[15]^ This invasive course needed to make an incision on the skin and carve the intercostal muscle if the large-bore chest tubes could be inserted into the pleural space, which may lead to possible complications like hemothorax and empyema. Comparing to the large-bore chest tubes, pigtail catheter with minimally invasive tubes had less pain and a smaller scar during the treatment and caused fewer complications. Based on these reasons, thus it appeared that duration of PC drainage less than chest tube treatment. In our study, the pooled duration of drainage was 5.57 days. Previous research showed that duration of secondary pneumothorax treatment in chest tube group was 11 ± 6 days,^[11]^ which was similar duration of 9.73 ± 5.96 days by Wei et al.^[2]^ It seemed that pigtail catheter drainage easier to conduct, had fewer procedures and traumas, and may be better tolerated in patients than the chest tube thoracostomy.

Some limitations should be addressed. First, heterogeneity was observed in the included studies. This could indicate differences in sample sizes, tube size, angle of puncture, and many other factors among the studies. Second, this was an one-arm study that was not compared with other traditional methods, and thus the pooled results are cautiously extrapolated. Third, most included studies were due to their retrospective nature and the relatively small sample size. Given the lack of evidence from more RCTs, well-designed RCTs of pigtail catheters for pneumothorax are needed.

In order to maximize the accuracy and reliability, we optimized search strategies. Meanwhile, to prevent from missing some important publications, we utilized broad search strategies. Subgroup analyses were also conducted according to the possible confounders of study design and race. Amazedly, subgroup analyses results were all consistent with the overall outcomes.

## 5 Conclusion

In conclusion, this meta-analysis demonstrated that pigtail catheters could be promising for pneumothorax. More large-scale RCTs studies are needed to assess the robustness of the findings.

## Author contributions

**Acquisition of data**: Ming Fang, Guilin Liu, Guoliang Luo, Tianyu Wu

**Analysis and interpretation of data**: Ming Fang, Guilin Liu

**Conceptualization:** Ming Fang.

**Critical revision of the manuscript for important intellectual content**: Guilin Liu, Guoliang Luo

**Data curation:** Ming Fang, Guilin Liu, Guoliang Luo, Tianyu Wu.

**Drafting of the manuscript**: Ming Fang

**Formal analysis:** Ming Fang, Guilin Liu, Guoliang Luo.

**Investigation:** Guoliang Luo.

**Methodology:** Guilin Liu, Guoliang Luo, Tianyu Wu.

**Software:** Guoliang Luo, Tianyu Wu.

**Statistical analysis**: Guilin Liu, Guoliang Luo

**Study concept and design**: Ming Fang

**Study supervision**: Ming Fang

**Supervision:** Ming Fang.

**Technical, or material support**: Guoliang Luo, Tianyu Wu

**Validation:** Ming Fang.

**Visualization:** Ming Fang.

**Writing – original draft:** Ming Fang.

**Writing – review & editing:** Ming Fang.
