# Does pigtail catheters relieve pneumothorax?: A PRISMA-compliant systematic review and meta-analysis: Erratum

**DOI:** 10.1097/MD.0000000000015270

**Published:** 2019-04-12

In the article, “Does pigtail catheters relieve pneumothorax?: A PRISMA-compliant systematic review and meta-analysis”,^[1]^ which appears in Volume 97, Issue 47 of *Medicine*, the phrase *“*In this systematic review and meta-analysis of 1124 cases of pneumothorax from 18 articles…*”* should be rephrased as following:” In this systematic review and meta-analysis of 1067 cases of pneumothorax from 16 articles…”
